# Correction: Analyzing the charge contributions of metal–organic framework derived nanosized cobalt nitride/carbon composites in asymmetrical supercapacitors

**DOI:** 10.1039/d5na90017d

**Published:** 2025-03-20

**Authors:** Vishal Shrivastav‡, Mansi‡, Prashant Dubey, Umesh K. Tiwari, Akash Deep, Wojciech Nogala, Shashank Sundriyal

**Affiliations:** a Institute of Physical Chemistry Polish Academy of Sciences Kasprzaka 44/52 01-224 Warsaw Poland vshrivastav@ichf.edu.pl wnogala@ichf.edu.pl; b CSIR-Central Scientific Instruments Organisation Sector 30-C Chandigarh 160030 India; c Advanced Carbon Products and Metrology Department, CSIR-National Physical Laboratory (CSIR-NPL) New Delhi 110012 India; d Institute of Nano Science and Technology (INST) Sector-81 Mohali 140306 Punjab India; e Regional Center of Advanced Technologies and Materials, The Czech Advanced Technology and Research Institute (CATRIN), Palacký University Olomouc Šlechtitelů 27 779 00 Olomouc Czech Republic shashank.sundriyal@upol.cz

## Abstract

Correction for ‘Analyzing the charge contributions of metal–organic framework derived nanosized cobalt nitride/carbon composites in asymmetrical supercapacitors’ by Vishal Shrivastav *et al.*, *Nanoscale Adv.*, 2024, **6**, 4219–4229, https://doi.org/10.1039/D4NA00291A.

The authors regret the omission of The Ministry of Education and Science, as an Acknowledgement for this research.

The corrected first sentence of the Acknowledgements is:

“Vishal Shrivastav wants to acknowledge the support from European Union’s Horizon 2020 research and innovation programme under the Marie Skłodowska-Curie grant agreement no. 847639 and from the Ministry of Education and Science.”

The Royal Society of Chemistry apologises for these errors and any consequent inconvenience to authors and readers.

